# Estimation of native and alkylated polycyclic aromatic hydrocarbons (PAHs) in seabirds from the south coast of the Baltic Sea

**DOI:** 10.1007/s11356-020-10653-y

**Published:** 2020-09-17

**Authors:** Ilona Waszak, Karolina Jonko-Sobuś, Agnieszka Ożarowska, Grzegorz Zaniewicz

**Affiliations:** 1 Department of Food and Environmental Chemistry, National Marine Fisheries Research Institute (NMFRI), 1 Kołłątaja Str, 81-332 Gdynia, Poland; 2 Avian Ecophysiology Unit, Department of Vertebrate Ecology and Zoology, University of Gdańsk, 59 Wita Stwosza Str, 80-308 Gdańsk, Poland

**Keywords:** Native PAHs, Alkylated PAHs, Greater scaup, Great crested grebe, Seabirds, Baltic Sea

## Abstract

Native and alkylated polycyclic aromatic hydrocarbons (PAHs) were examined in the muscle, liver, kidneys, and lungs of greater scaup (*Aythya marila*) and great crested grebe (*Podiceps cristatus*). Both species showed differences in distribution and profiles among the tissues. The highest ƩPAH concentrations were in the scaup lungs and the grebe kidneys (20 and 19 ng g^−1^ wet weight, respectively). Alkylated derivatives were in the range of 61 to 77% of ΣPAHs in both species. Differences in PAH profiles between birds, analyzed by PCA, were probably results of metabolic capability, not various diet. In comparison to available data from other regions of the world, PAH levels in investigated species are relatively low and do not pose a threat to birds themselves.

**Electronic supplementary material:**

The online version of this article (10.1007/s11356-020-10653-y) contains supplementary material, which is available to authorized users.

## Introduction

Polycyclic aromatic hydrocarbons (PAHs) are ubiquitous and well-known environmental pollutants which have attracted a lot of scientific interest due to tendency to bioaccumulate and biomagnify in the food web. Furthermore, some of them are genotoxic, carcinogenic, and mutagenic; as a consequence, they can lead to many disorders such as reproductive dysfunction, increased susceptibility to diseases or other stresses, and changes in the behavior of animals and humans (Custer et al. 2000). PAHs never occur in the environment as individual compounds, but as a mixture of many components, including hundreds of parent PAHs and their alkylated derivatives. In contrast to unsubstituted compounds, alkylated PAHs (aPAHs) are more resistant to degradation and therefore, they are longer present in the environment. Little is known about the toxic properties of aPAHs, although there are reports that they may pose a greater risk to organisms than their parent counterparts (Marvanová et al. 2008). For this reason, it is pointed out that not only PAHs but also their alkylated derivatives (EPA 2003; 2004) should be included in the environmental risk assessment.

Distribution and concentrations of PAHs in various environmental components depend on many factors. The type of source and origin determines the nature and quantity of compounds released into the environment. Although some PAHs arise from natural processes, such as forest, grass, and bush fires, volcanic eruptions, and production by microorganisms from biogenic precursors, emissions from anthropogenic activities, including combustion and processes related to use of crude oil, are considered predominant in the environment (Boitsov et al. 2009; Dalsøren et al. 2007). PAHs are widespread atmospheric pollutants (Zhou et al. 2005). The solubility of PAHs in water is low and decreases with increasing molecular weight; therefore, the concentrations of dissolved PAHs in water are very low. PAHs tend to associate with solid particles and fall to the bottom (Götz et al. 1998). Due to their stability, PAHs remain in sediments for many years, posing a threat to dwelling organisms. Therefore, monitoring programs of PAHs in the Baltic region mostly include research of the sediments and benthos (Lubecki and Kowalewska 2012; Namieśnik et al. 2008; Waszak et al. 2019). There is relatively little data on PAHs in seabirds. Most bird species occupy a high position in the trophic pyramids of the Baltic Sea (predators), which results in accumulation of the largest amounts of harmful substances in their tissues and organs. Therefore, birds are considered to be good bioindicators of persistent environmental pollutants such as PCBs and PBDEs, or heavy metals (Badzinski et al. 2009; Jaspers et al. 2006; Tomza-Marciniak et al. 2019). However, in the case of PAHs, which are more prone to be degraded, the use of seabirds as biomonitors is still unclear and scarcely explored. Avian exposure to PAHs probably occurs mainly via oral intake of contaminated invertebrates, plants, and small fish (Kayal and Conell, 1995), but the high concentrations of PAHs in the lungs of some investigated species (Zhang et al. 2015) can also indicate inhalation of polluted air as important pathway for these compounds. Birds, like other vertebrates, generally display high oxidative P-450 enzyme activity and can quickly metabolize and easily excrete most of consumed PAHs (Näf et al. 1992; Troisi et al. 2006; Verbrugge et al. 2001). However, it has been documented that there is a relationship between the presence of PAHs in bird tissues and petroleum contamination of surface water (Custer et al. 2000). The yellow-legged gull (*Larus michahellis*) was suggested as a good indicator of PAH contamination after comparing levels from colonies polluted by the Prestige oil spill with non-affected areas (Pérez et al. 2008).

In the present study, the PAH concentrations were measured in two species of seabirds, greater scaup (*Aythya marila*) and great crested grebe (*Podiceps cristatus*), from the two areas of south coast of the Baltic Sea, Pomeranian Bay (PB) and Szczecin Lagoon (SL). These two species of birds differ in diet. The scaup’s basic food is benthos, represented by zebra mussel (*Dreissena polymorpha*) in the SL area (Marchowski et al. 2015), and by sand gaper (*Mya arenaria*), blue mussel (*Mytilus edulis*), and baltic clam (*Macoma balthica*) in the maritime zone (PB) (Mendel et al. 2008; Stempniewicz and Meissner 1999). The grebe favors a fish-based diet (Ulenaers and Vessem 1994), where smelt (*Osmerus eperlanus*) predominates and some benthic fishes, e.g., flounder (*Platichthys flesus*), appear less frequently (Morkune et al. 2016; Piersma et al. 1988). The area from which the scaup and grebe samples were collected is one of the most important wintering location for many native and arctic species of birds, where coastal waters are characterized by an abundant food supply. However, this is an area under strong anthropogenic pressure, which is influenced by maritime economy with some important sea routes for ships, numerous ports and repair shipyards, the fuel and energy industry, and mining (crude oil). In the aspect of these issues, the present study investigates the levels, distribution, and potential sources of PAHs in the seabirds from the south coast of the Baltic Sea, to gain a better understanding of the interspecies differences and their routes of exposure.

## Materials and methods

### Sampling strategy

**Fig. 1:**
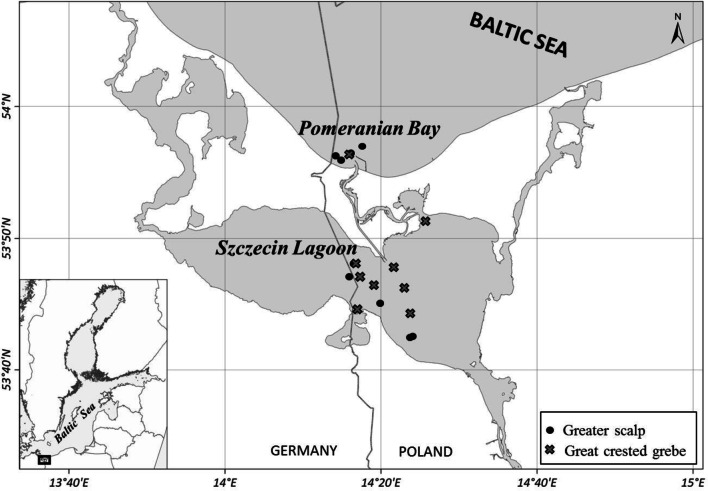
The bird sampling collections in the southern Baltic Sea

Eleven specimens of the two bird species were selected for the study (Table 1). The examination concerned only individuals which were found dead in fishing nets. None of them was killed for the purpose of performing this study. All birds were obtained from the area covering the part of Pomeranian Bay, PB (53°55–56′N, 14°14–19′E), and the area of Szczecin Lagoon, SL (53°42–51′N, 14°16–26′E), during winter 2014–2015 (Fig. 1). The biological parameters of the birds, i.e., physical condition, gender (male, M vs female, F), age, and body mass, were evaluated. The 2 M and 3F of scaup and only 1F of grebe were obtained from PM, while 3 M and 3F of scaup and 6 M and 4F of grebe were from SL. Only specimens aged 2 years (or slightly above) in good condition were taken to the section. The breast muscles, liver, kidneys, and lungs were freeze-dried and stored at − 20 °C until analysis.

**Table 1:** Biological characteristics of birds from the southern Baltic Sea (values are mean with the range of results)

Species	Gender	*N*^1^	Body mass (g)	Lipids (%)^2^			
				Muscle	Liver	Kidneys	Lungs
Greater scaup (*Aythya marila*)	Male	5	1312 (1128–1460)	3.6 (2.9–5.2)	3.2 (2.5–4.1)	5.0 (2.7–6.9)	2.3 (1.7–3.4)
	Female	6	1156 (1016–1303)	3.8 (3.3–4.3)	3.1 (2.7–3.4)	3.1 (2.1–5.8)	1.7 (1.4–2.3)
Great crested grebe (*Podiceps cristatus*)	Male	5	1429 (1204–1551)	5.0 (2.2–8.3)	3.6 (2.5–5.5)	3.9 (3.4–4.9)	2.5 (1.9–3.3)
	Female	6	1237 (1104–1405)	4.5 (3.2–6.4)	3.2 (2.9–5.6)	3.5 (2.9–4.9)	2.4 (1.5–3.5)

^1^The number of specimens. ^2^The lipid content (%) in wet weight of tissue

### Preparation of samples for analysis

For PAH analyses, the all tissues were extracted with a mixture of n-hexane: dichloromethane (DCM) (1:1, *v*/v) using a fully automated ASE 350 system (Dionex, Sunnyvale, CA, USA). Ten percent (*v*/v) of the extract was taken for the lipid content determination. The volume containing 0.3 g of lipid from the remaining part of the extract was dissolved in 10 ml of n-hexane and supplemented with surrogate standards (SS, acenaphthene-d_10_, chrysene-d_12_, naphthalene-d_8_, phenanthrene-d_10_, and perylene-d_12_). After adding 10 ml of KOH (0.5 M solution in MeOH:H_2_O, 1:1, v/v), the mixture was gently mixed and then centrifuged at 2000 rpm for 40 min. After centrifugation, the n-hexane layer (containing the compounds of interest) was transferred into a flask and the separation procedure was repeated two more times with aliquots of 10 ml n-hexane. The obtained extracts were combined and concentrated using a rotatory evaporator to about 1 ml, and then loaded on a glass column (5 g of 5% deactivated silica gel), and the compounds were eluted with 100 ml of n-hexane:DCM (95:5%, v/v). This extract was concentrated, solvent exchanged to isooctane, transferred to a glass vial, and reconstituted using a gentle nitrogen stream to a final volume of 0.2 ml. The lipid content in tissues was determined gravimetrically on an aliquot of the extract for each sample.

### GC-MS analysis

The method of analysis was based on the Waszak et al. (2019), Ibadov and Suleymanov (2015), and Martinez et al. (2004) guidelines with some modifications. Analyses were performed using gas chromatography–mass spectrometry (GC–MS) with a 30-m DB-5MS column. The sample injection, transfer line, and ion source temperatures were 290 °C, 280 °C, and 200 °C, respectively. The column temperature was initially held at 40 °C for 1 min and gradually raised to 120 °C at the rate of 25 °C min^−1^, then to 160 °C at 10 °C min^−1^, and finally to 300 °C at 5 °C min^−1^, and held for 15 min. The total analysis time was 51.2 min. Helium, as the carrier gas, had a constant flow rate of 1 ml min^−1^. Mass spectrometry was performed using electron ionization (EI) and selective ion monitoring (SIM) mode. The compounds were identified based on retention times and mass spectra. Their concentrations were calculated referring to isotope-labeled standards (SS) recovery (SupData; Table 1). The range of retention times of PAH derivatives was established based on crude oil sample analysis (e.g., Diesel Fuel #2 dissolved in DCM). The following compounds were analyzed: 16 unsubstituted PAHs recommended by the US EPA: acenaphthene (Ace), acenaphthylene (Acy), anthracene (Ant), benzo[a]anthracene (B[a]A), benzo[b]fluoranthene (B[b]F), benzo[k]fluoranthene (B[k]F), benzo[ghi]perylene (B[ghi]P), benzo[a]pyrene (B[a]P), chrysene (Chr), dibenzo[a,h]anthracene (DB[ah]A), fluoranthene (Flt), fluorene (Flu), indeno[1,2,3-cd]pyrene (I[cd]P), naphthalene (Naph), phenanthrene (Phe), and pyrene (Pyr), and alkylated derivatives of naphthalene (C1-, C2-, C3-Naph), phenanthrene and anthracene (C1- and C2-Phe/Ant), and dibenzothiophene (C1- and C2-DBT). The concentrations of individual PAHs were summed as follows: Σ_16_PAHs (16 unsubstituted PAHs), Σ3-ring PAHs (Ace, Acy, Flu, Phe, and Ant), Σ4-ring PAHs (Flt, Pyr, B[a]A, and Chr), Σ5-ring PAHs (B[b]F, B[k]F, B[a]P, and DB[ah]A), and Σ6-ring PAHs (I[cd]P and B[ghi]P), ΣaPAHs (C1-, C2-, and C3-Naph; C1-, C2-Phe/Ant, and C1-, C2-DBT), ΣPAHs (Σ_16_PAHs and ΣaPAHs). The limit of detection (LOD) for individual PAHs was 0.01 ng g^−1^ wet weight (ww) (Table 2). Quality control included procedural blanks (SupData; Table 2), duplicates, standard analysis, and recovery of surrogate standards added to each sample and blanks. The average recovery of SS was from 67 to 166% (SupData; Table 3). The PAH concentrations in tissues were blank- and SS-recovery-corrected.

**Table 2:** Concentrations of native and alkylated PAHs in birds from the southern Baltic Sea (ng g^-1^ ww; mean and standard deviations)

	Greater scaup				Great crested grebe			
	Muscle	Liver	Kidneys	Lungs	Muscle	Liver	Kidneys	Lungs
16PAHs								
Naph	0.2 ± 0.1	0.7 ± 0.5	1.0 ± 0.2	4.7 ± 1.2	0.8 ± 0.3	1.0 ± 0.4	2.9 ± 2.0	3.7 ± 2.0
Acy	0.04 ± 0.01	0.05 ± 0.04	0.07 ± 0.03	0.03 ± 0.02	0.1 ± .0.06	0.04 ± 0.01	0.2 ± 0.1	0.04 ± 0.03
Ace	0.05 ± 0.01	0.09 ± 0.03	0.2 ± 0.1	0.1 ± 0.05	0.1 ± 0.03	0.09 ± 0.03	0.2 ± 0.01	0.2 ± 0.1
Flu	0.2 ± 0.1	0.2 ± 0.1	0.3 ± 0.2	0.2 ± 0.1	0.3 ± 0.1	0.1 ± 0.06	0.5 ± 0.4	0.2 ± 0.04
Phe	0.8 ± 0.3	0.8 ± 0.7	0.8 ± 0.4	0.7 ± 0.5	0.9 ± 0.6	0.5 ± 0.3	2.3 ± 1.5	0.7 ± 0.2
Ant	0.03 ± 0.01	0.03 ± 0.02	0.02 ± 0.01	0.02 ± 0.01	0.06 ± 0.01	0.04 ± 0.02	0.04 ± 0.01	0.02 ± 0.01
Flt	0.3 ± 0.1	0.3 ± 0.2	0.3 ± 0.1	0.2 ± 0.1	0.2 ± 0.1	0.3 ± 0.2	0.6 ± 0.4	0.2 ± 0.1
Pyr	0.2 ± 0.1	0.2 ± 0.1	0.3 ± 0.2	0.2 ± 0.1	0.2 ± 0.03	0.2 ± 0.1	0.6 ± 0.3	0.2 ± 0.1
B(a)A	nd	0.03 ± 0.1	nd	0.01 ± 0.003	0.05 ± 0.03	0.07 ± 0.02	0.08 ± 0.02	0.01 ± 0.003
Chr	0.04 ± 0.01	0.08 ± 0.05	0.05 ± 0.02	0.06 ± 0.02	0.07 ± 0.05	0.1 ± 0.03	0.04 ± 0.01	0.02 ± 0.01
B(b)F	nd	nd	0.04 ± 0.01	0.05 ± 0.01	0.06 ± 0.02	0.4 ± 0.1	0.08 ± 0.03	0.04 ± 0.01
B(k)F	nd	nd	nd	nd	nd	nd	0.2 ± 0.1	nd
B(a)P	nd	nd	nd	nd	0.2 ± 0.1	nd	0.1 ± 0.05	nd
I(cd)P	0.03 ± 0.01	0.04 ± 0.02	0.07 ± 0.03	0.02 ± 0.01	0.06 ± 0.01	0.3 ± 0.2	0.06 ± 0.04	0.01 ± 0.002
DB(ah)A	0.04 ± 0.01	0.03 ± 0.01	0.1 ± 0.03	nd	0.08 ± 0.03	0.3 ± 0.1	0.07 ± 0.03	nd
B(ghi)P	nd	0.03 ± 0.02	nd	nd	0.05 ± 0.01	0.2 ± 0.1	0.03 ± 0.01	nd
aPAHs								
C1-Naph	0.7 ± 0.2	3.0 ± 0.8	2.0 ± 0.7	8.5 ± 2.2	1.8 ± 0.6	1.9 ± 0.9	3.0 ± 1.0	4.5 ± 2.2
C2-Naph	0.8 ± 0.5	1.4 ± 0.8	1.5 ± 0.7	2.1 ± 1.0	1.3 ± 0.5	1.3 ± 0.6	2.5 ± 1.6	1.4 ± 0.5
C3-Naph	0.8 ± 0.3	1.5 ± 1.0	1.0 ± 0.5	1.5 ± 1.1	1.5 ± 0.7	1.1 ± 0.4	2.4 ± 1.0	1.0 ± 0.4
C1-Phe/Ant	0.9 ± 0.4	1.1 ± 0.8	1.9 ± 0.9	0.5 ± 0.3	2.5 ± 1.1	0.9 ± 0.5	2.3 ± 1.6	0.6 ± 0.2
C2-Phe/Ant	0.7 ± 0.3	1.3 ± 0.8	0.7 ± 0.5	0.4 ± 0.1	2.0 ± 0.9	1.1 ± 0.7	2.2 ± 1.5	0.5 ± 0.1
C1-DBT	0.2 ± 0.1	0.4 ± 0.3	0.3 ± 0.2	0.2 ± 0.1	0.4 ± 0.1	0.5 ± 0.3	0.4 ± 0.3	0.3 ± 0.1
C2-DBT	0.3 ± 0.2	1.3 ± 0.5	0.4 ± 0.3	0.1 ± 0.03	1.0 ± 0.6	0.8 ± 0.5	0.6 ± 0.3	0.2 ± 0.1
Ʃ3-ring PAHs	1.1 ± 0.5	1.2 ± 0.7	1.3 ± 0.6	1.1 ± 0.5	1.5 ± 0.9	0.8 ± 0.3	3.2 ± 2.2	1.1 ± 0.5
Ʃ4-ring PAHs	0.4 ± 0.3	0.6 ± 0.3	0.6 ± 0.3^A^	0.5 ± 0.1	0.4 ± 0.1	0.5 ± 0.2	1.2 ± 0.8^B^	0.4 ± 0.2
Ʃ5-ring PAHs	0.01 ± 0.008^A^	0.01 ± 0.006^A^	0.02 ± 0.01^A^	0.05 ± 0.03	0.3 ± 0.2^B^	0.3 ± 0.2^B^	0.3 ± 0.1^B^	0.04 ± 0.01^a^
Ʃ6-ring PAHs	0.01 ± 0.006^A^	0.03 ± 0.01^A^	0.01 ± 0.006	0.02 ± 0.005	0.1 ± 0.04^a,B^	0.5 ± 0.3^b,B^	0.02 ± 0.01^ac^	0.02 ± 0.01^c^
Ʃ_16_PAHs^1^	1.5 ± 0.9^a^	2.5 ± 2.0^ab^	2.6 ± 1.8^ab,A^	6.4 ± 2.1^b^	2.7 ± 1.9^a^	2.8 ± 1.9^a^	7.3 ± 5.5^b,B^	5.2 ± 3.1^ab^
ƩaPAHs^2^	4.0 ± 1.5^a,A^	9.1 ± 4.3^ab^	7.2 ± 2.5^a,A^	13 ± 4.1^b^	8.9 ± 5.3^B^	7.5 ± 3.5	11 ± 3^B^	8.4 ± 3.7
ƩPAHs^3^	5.5 ± 0.8^a,A^	12 ± 4.1^ab^	9.3 ± 3.0^a,A^	20 ± 3.6^b^	12 ± 5.0^a,B^	10 ± 7.0^a^	19 ± 7.1^b,B^	14 ± 6.2^ab^
ƩaPAHs (%)^4^	73	76	77	66	77	73	61	62
Ʃ_16_PAHs^5^	41 ± 9.1^a^	80 ± 50^ab^	79 ± 29^b,A^	380 ± 101^c,A^	56 ± 13^a^	86 ± 52^ab^	210 ± 152^bc,B^	214 ± 129^c,B^
ƩaPAHs^5^	80 ± 32^a,A^	322 ± 95^b^	153 ± 65^a,A^	779 ± 479^c,A^	176 ± 70^a,B^	230 ± 92^ab^	366 ± 93^b,B^	381 ± 142^b,B^
ƩPAHs^5^	121 ± 35^a,A^	402 ± 122^b^	232 ± 84^c,A^	1159 ± 236^d,A^	232 ± 76^a,B^	315 ± 210^ab^	573 ± 221^b,B^	601 ± 252^b,B^

^1^The sum of 16 native PAHs. ^2^The sum of alkylated PAHs derivatives. ^3^The sum of native and alkylated PAHs. ^4^ƩaPAHs expressed as the percentage (%) of ƩPAHs. ^5^Concentration in ng g^-1^ lipid. ^a–d^Significant intraspecies differences among PAH concentrations in tissues. ^A,B^Significant interspecies differences in PAHs concentrations. *nd*, the concentration below the detection limit

**Table 3:** Native and alkylated PAH concentrations in seabirds around the word (ng g^−1^ ww, mean and the range of values)

Site	Year	Species	Tissue	PAHs group	Concentrations	References
South cost of the Baltic Sea	2014-2015	Greater scaup (*Aythya marila*)	Muscle Liver Kidneys Lungs	∑_16_PAHs, C1-C3-Naph, C1-C2-Phe/Ant, C1-C2-DBT	4.5 (2.0-6.7) 11 (6.4-16) 7.3 (5.1-11) 20 (13-23)	This study
		Great crested grebe (*Podiceps cristatus*)	Muscle Liver Kidneys Lungs		12 (6.8-17) 10 (7.2-17) 19 (12-26) 14 (9.8-20)	
Gulf of Gdańsk, Baltic Sea	2010-2012	Herring gulls (*Larus argentatus*)	Lungs	∑_5_PAHs	4.6-115*	Falkowska et al. (2017)
Northern Baltic proper	1990	Common eider (Somateria mollissima)	Liver Kidney	Σ_16_PAHs	24 18	Näf et. al (1992)
Brisbane River estuary (Australia)	1988	Pelican (*Pelecanus conspicillatus*) Silver gull (*Larus novaehollandiae*)	Muscle	Σ_8_PAHs, C2-Naph	75 85	Kayal and Connell (1995)
Indiana Harbor Canal (East Chicago, USA)	1994	Lesser scaup (*Aythya affinis*)	Carcasses	Naph, Phe, B(a)P, C1-Naph	115 (95-135)	Custer et al. (2000)
East coast of England	2001-2002	Common guillemots (*Uria aalge*)	Liver	Σ_10_PAHs	245 (43-972)	Troisi et. al (2006)
Northeast Atlantic Mediterranean Sea	2003-2007	5 species of Procellariiformes	Liver	Σ_15_PAHs	24 (3.3-66) 5.8 (1.5-10)	Roscales et. al (2011)

*Concentration in ng g^-1^ dry weight.

### Statistical analysis

Statistical analysis of the data was performed using the Statistica 10.0 (StatSoft Inc., Tulsa, OK, USA). Prior to the analyses, the distribution of the data was determined using the Shapiro-Wilk *W* test. Differences in biological parameters between M and F of each specimens, as well as between scaups and grebes, were examined with either *t* test or Mann-Whitney test, depending on whether or not assumption of normality and homogeneity of variance was met. These both statistic tests was also used to examine PAH concentrations in tissues (Σ3-, 4-, 5-, and 6-ring PAHs, Σ_16_PAHs, ΣaPAHs, and ΣPAHs) between both species. Intraspecies differences in the PAH concentrations among the tissues were examined with either ANOVA (Tukey test with HSD) or Kruskal-Wallis test. If more than 50% of the observations were with PAH levels below LOD, no statistical analyses were conducted. For the other observations with PAH concentrations below LOD, a value of LOD/2 was assigned for the statistical analysis. Relationships between biological parameters (body mass and tissue lipid content) and PAH concentrations were evaluated using regression analysis in a general linear model (GLM) or the nonparametric Spearman test. The birds did not differ sexually due to biological parameters and PAH concentrations within each species (Table 1); therefore, males and females were consider as a one group (scaups or grebes) in this study. All results were given as means and their standard deviations.

## Results and discussion

To our knowledge, this is the first study in which PAH concentrations have been evaluated in birds from the Pomeranian Bay and Szczecin Lagoon on the south cost of the Baltic Sea. The present study results suggest that PAHs in bird, despite of the metabolic capacity, are found in their tissues. The PAH levels differed between both analyzed species and among tissues within the same species as well.

### Greater scaup

**Fig. 2:**
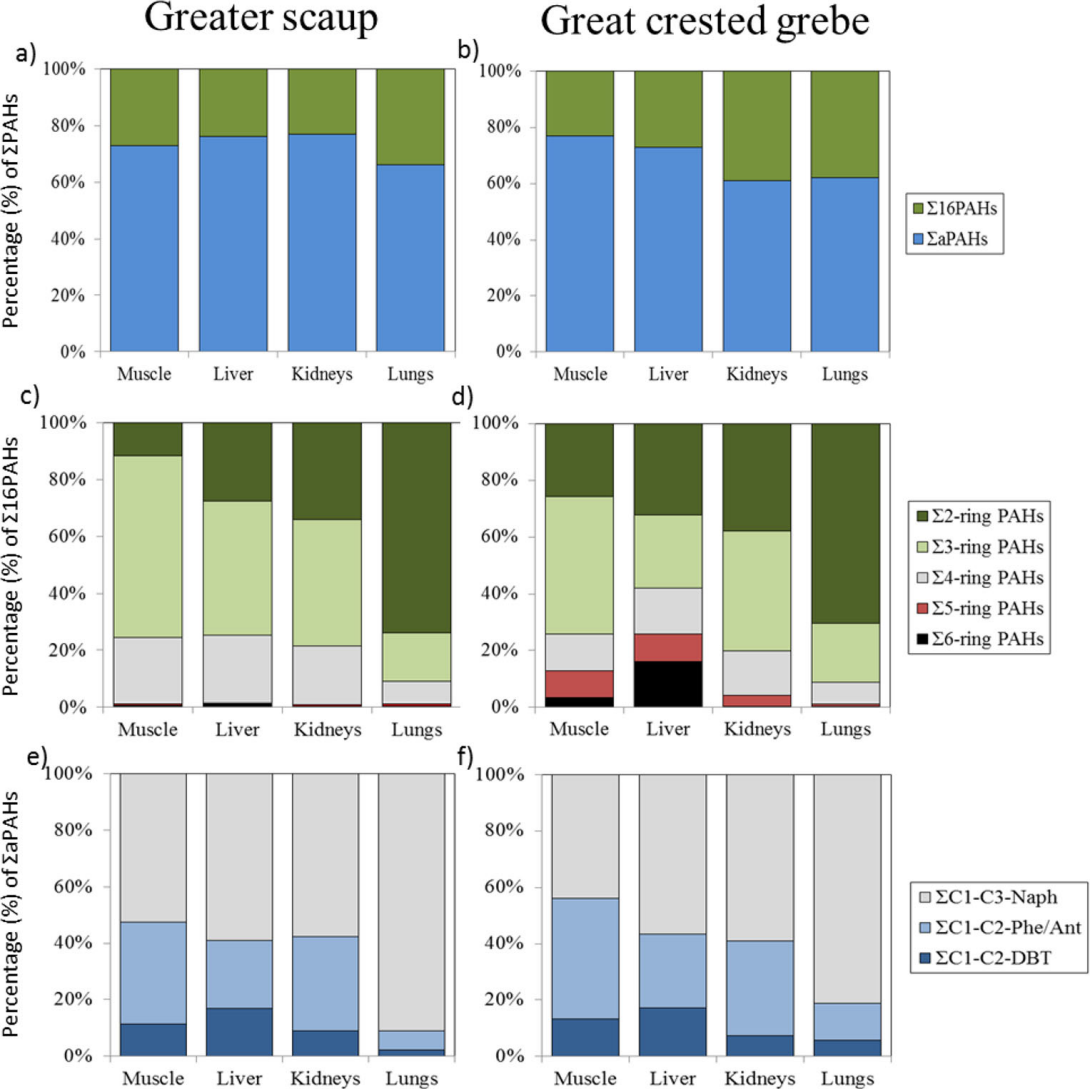
Tissue composition of ΣPAHs (**a**, **b**), Σ_16_PAHs (**c**, **d**), and ΣaPAHs (**e**, **f**) in birds

The mean concentration of Σ_16_PAHs, ΣaPAHs, and ΣPAHs in scaup differed significantly among tissues and based on wet weight, it was in the range of 1.5–6.4, 4.0–13, and 5.5–20 ng g^−1^, respectively, whereas lipid-normalized concentrations were in the range of 41–380, 80–779, and121–1159 ng g^−1^, respectively (Table 2). ΣaPAHs constituted 66–77% of ΣPAHs and had the greatest contribution in the liver and kidneys (Fig. 2a). The highest wet weight and lipid-normalized concentration of all analyzed group of compounds occurred in the lungs, followed by the liver and kidneys. Compared with the literature, the Σ_16_PAH and ΣPAH levels in all the tissues were lower than those previously reported in birds from other regions, with exception of the similar levels of Σ_16_PAHs in the scaup liver and those reported in Procellariiformes from the Mediterranean Sea. In relation to contaminated area, the concentrations were up to an order of magnitude lower than those observed in the liver of oiled common guillemots (*Uria aalge*) stranded on the east coast of England (Table 3). Like the PAH concentrations, their composition varied significantly among the scaup tissues as well. Among native compounds, the dominants in the muscle, liver, and kidneys were 3-ring PAHs with contribution of 47–73% of Σ_16_PAHs, whereas in the lungs 2-ring PAHs dominated (74% of Σ_16_PAHs; Fig. 2c). The dominant individual compound in the muscle and liver was Phe (54% and 30% of Σ_16_PAHs, respectively), but in the kidneys and lungs, it was Naph (37% and 73% of Σ_16_PAHs, respectively) followed by Phe (31% and 11% of Σ_16_PAHs, respectively) (Table 2). Among alkylated compounds, C1–C3-Naph dominated in all tissues (58–93% of ΣaPAHs), but it was significantly high fraction of them in the lungs (Fig. 2e). This high proportion of Naph and its alkylated homologs in relation to other analyzed compounds in the lungs resulted in this organ characterized by the highest ΣPAH concentrations among all examined tissues. Although in most of the literature, PAH profiles in seabird differed among species and their tissues, Roscales et al. (2011) found the similar Σ_16_PAHs profile in the liver of two species of petrel (*Bulweria bulwerii* and *Pelagodroma marina*) from Atlantic, in which 3-ring PAHs dominated and 5–6-ring PAHs had the smallest contribution. The same authors reported that the most abundant compound in the petrel liver was Flt, followed by Naph. With regard to aPAH profiles, so far the less frequently analyzed, alkyl naphthalenes (C1–C4-Naph) were stated as dominants in fat tissues of penguin (*Pygoscelis* sp.) and skula (*Catharacta antarctica*) from King George Island (Antarctica) (Taniguchi et al. 2009). Regression analysis of wet weight concentration of Σ_16_PAHs in each tissue versus scaup body mass indicated no significant relationships between all these variables. However, Σ_16_PAH concentration was significantly related to lipid content in the muscle and lungs (*p* < 0.03), but not in the liver and kidneys. For ΣaPAHs, the concentration only in the muscle was significantly related to bird body mass (*p* = 0.004) and not correlated with lipid content in any tissue. In the literature reviewed, no previous studies were found on the relationship between PAH content in aquatic birds and their body mass (length) or lipid content.

### Great crested grebe

The wet weight concentration of Σ_16_PAHs, ΣaPAHs, and ΣPAHs in grebe tissues, in the range of 2.2–7.3, 7.5–11, and 10–19 ng g^−1^, respectively, as well as the lipid-based concentration in the range of 56–214, 176–381, and 232–601 ng g^−1^, respectively, showed significant differences (Table 2). ΣaPAHs constituted 61–77% of ΣPAH levels and had the greatest contribution in the muscle (Fig. 2a). The highest PAH wet weight concentrations occurred in the kidneys, although they were nearly two times lower than those reported in the kidneys of common eider from the northern Baltic (Table 3). Similarly, the PAH wet weight levels in the other grebe tissues were relatively lower than in different bird species from the other sites documented in the literatures. PAH levels in grebe expressed in lipid content were the highest in the lungs and kidneys (Table 2). Composition of parent PAHs differed significantly among tissues with the greatest contribution of 3-ring PAHs in the muscle and kidneys (42–56% of Σ_16_PAHs) and 2-ring PAHs in the liver and lungs (36–70% of Σ_16_PAHs) (Fig. 2d). There were also visible differences in 6-ring PAH share, in which contribution was relatively high in the liver (18% of Σ_16_PAHs) compared with that in other tissues (less than 2%). The dominant compounds in the muscle and kidneys were Naph and Phe (30–40% of Σ_16_PAHs), but in the liver and lungs, it was Naph (36% and 71% of Σ_16_PAHs, respectively) (Table 2). In comparison to available literature data, among five bird species, only the liver of piscivorous shearwater (*Calonectris borealis*) from the Atlantic had the similar Σ_16_PAH profiles, in which 2-ring PAHs dominated and Naph was the most abundant compound (Roscales et al. 2011). Furthermore, 5- and 6-ring PAHs had the same contribution in the profiles (about 5% of Σ_16_PAHs) in contrast to the other species. Alkylated PAH profiles in all tissues were fairly similar with the dominant group being C1–C3-Naph (52–83% of ΣaPAHs); however, the lungs showed relatively the greatest percentage of alkylated naphthalenes in comparison with the other tissues (Fig. 2f). There were no statistically significant relationships between Σ_16_PAH and ΣaPAH wet weight concentration in the tested tissues and grebe body mass, but these two groups of compounds were related to lipid content in the muscle (*p* < 0.02), not in other tissues.

### Interspecies differences

**Fig. 3:**
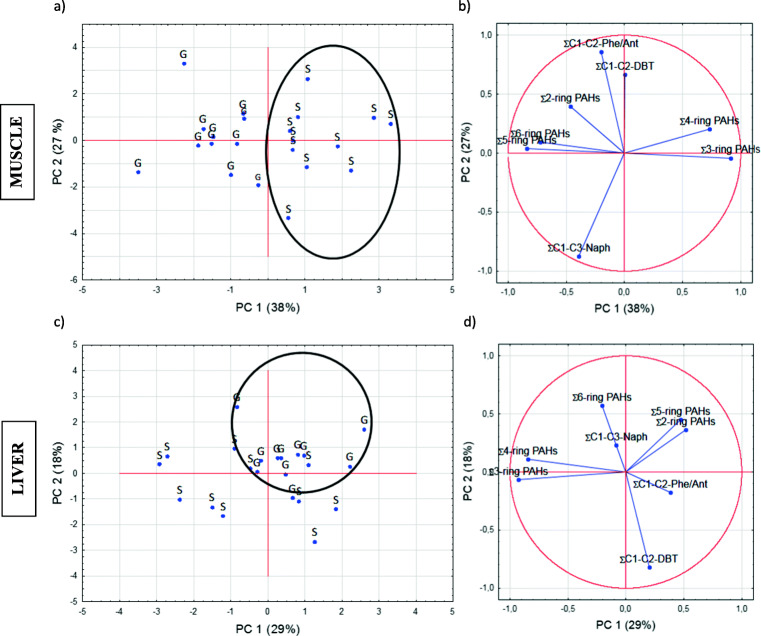
PCA analysis scores (**a**, **c**) and loadings (**b**, **d**) of PAH native and alkylated groups expressed as the percentage of ΣPAHs in scaup (S) and grebe (G) muscle and liver

The examined species of birds demonstrated similar general wet weight levels of PAHs in their bodies (Table 2), indicating the highest values in the kidneys and lungs, which is usually observed in seabirds (Zhang et al. 2015). This may be due to the fact that the bird kidneys occupy up to about 20% of their body weight and play a key role in xenobiotic metabolism, whereas bird respiratory system is the largest area of the organism’s interaction with airborne pollutants (Sanderfoot and Holloway 2017; Zhang et al. 2015). Our research showed some interspecies differences in PAH concentrations in bird tissues. Σ_16_PAH, ΣaPAH, and ΣPAH levels in the kidneys were about 1.5–3 times greater in grebe than in scaup and these differences were statistically significant. Whereas concentrations of ΣaPAHs and ΣPAHs in the lungs were found to be 1.5-fold greater in scaup than in grebe, these differences were not statistically significant. Σ_16_PAH, ΣaPAH, and ΣPAH levels in the muscle were nearly twofold greater in grebe than in scaup, and these differences for ΣaPAH and ΣPAH concentrations were statistically significant. PAH levels in the liver did not differ between species. The same interspecies differences were observed for lipid-normalized concentrations of PAHs in tissues, except for the lungs, where the levels showed statistically significant differences, being about twofold greater in scaup than in grebe. The most likely cause for the occurrence of statistically insignificant differences in PAH levels between the birds was the large range of the obtained results, which in turn may be to the results of a relatively small number of individuals included in the study. However, it was not possible to obtain a larger number of the species in the time available. There were no significant differences in gender, body mass, and tissue lipid content between the birds (Table 1); therefore, these biological parameters did not affect interspecies differences in PAH levels. Furthermore, the birds could move between the two areas studied and it was considered that the regional differences are not significant. Whereas, it is possible that birds whose food base consists mostly of fish will be characterized by lower residues of PAHs in comparison with birds which feed on invertebrates (Kayal and Connell 1995; Lebedev et al. 1998). However, although scaup and grebe feed on different food types, no statistically significant differences were observed in the case of ƩPAH levels in the liver of both species in the present study. Liver as the target tissue had been suggested to provide information regarding short-term exposure to PAHs in vertebrates due to its large and rapid detoxification capability (Custer et al., 2001; Hellou 1996; Roscales et al. 2011). Another explanation of differences between PAH concentrations in bird tissues could be metabolic capabilities. This can result in differences in PAH profiles between species (Fig. 2). To compare PAH composition in scaup and grebe tissues, the principal component analysis (PCA) was done (Fig. 3). PCA was based on a percentage share of native and alkylated groups of compounds in ΣPAH concentration in tissues. The data was auto-scaled with standard deviation set to 1 and mean value to 0. Principal components (PCs) were extracted based on eigenvalues greater than 1. Figure 3 graphically presents results of PCA for differences in PAH composition in the muscle and liver between birds. PCA graphic presentation for kidneys and lungs indicated that PAH composition was not different for both species, and therefore, it was not presented in the study. PCA for the muscle (Fig. 3a, b) identified two groups of birds, one included only grebe (G) and second with scaup (S) specimens. The first group, including grebe specimens (G), was linked with loading of alkylated and native Σ2-ring PAHs, Σ5-ring PAHs, and Σ6-ring PAHs. The second group, including scaup specimens, was associated with high positive loading of Σ3- and Σ4-ring PAHs. PCA for liver distinguished between the two groups of birds, i.e., scaup and grebe, but in less explicit way (Fig. 3c, d). Scaup group was clearly linked with Σ3- and Σ4-ring PAHs, and ΣC1–C2 DBT, but grebe group showed loadings of Σ2-, Σ5-, and Σ6-ring PAHs, ΣC1–C3 Naph, and ΣC1–C2-Phe/Ant. The most visible difference between both species in the muscle and liver profiles is in Σ2- and Σ5–6-ring PAH contribution, and indicates other metabolic abilities of birds. All birds are equipped with a well-developed mixed-function oxidase (MFO) system that facilitates biotransformation and detoxification of exogenous chemicals, including PAHs (Albers and Loughlin 2003). The lower levels of Σ5–6-ring PAHs in the scaup than in the grebe tissues suggest the first species has a higher biotransformation capacity than the second. Roscales et al. (2011) reported that the low presence of high molecular weight PAHs in the profile of petrel liver comparing to shearwaters analyzed in their studies may be explained by the greater capability of petrels to metabolize the larger PAH compounds. Similarly, according to Troisi et al. (2006), the absence of B(a)P from guillemot livers suggests this species has a high capacity for CYP4501A1-mediated B(a)P metabolism.

### PAH sources and risk assessment

The specific pattern of PAH concentrations in the environment depends on the source of these compounds’ contamination. The ratio of levels of PAHs with low molecular weight to that with high molecular weight is commonly used to distinguish between pyrolysis sources from petroleum or incomplete combustion sources of pollution (Fernandes et al. 1997). The concentrations of Σ2–3-ring PAHs constituting more than 50% of ΣPAHs indicate a dominance of petroleum pollution and incomplete combustion, while levels of Σ4–6-ring PAHs being of > 50% of ΣPAHs suggest a dominance of pyrolysis source. In the present study, the PAH composition in the tissues of both species indicates that petroleum and incomplete combustion can be the major sources of contamination. Similar data on the PAH sources in seabirds are widely reported (Custer et al. 2000; Taniguchi et al. 2009; Troisi et al. 2006). However, it should be noted that a different exposure to these contaminants through diet or different metabolic capabilities among species may alter the ratios of the PAHs that are pointing out different sources.

Avian exposure to PAHs seems to be related to contaminated diet, water, and air through inhalation and feather preening (Fernie et al. 2018). This study, in line with the majority of available reports (Kayal and Connell 1995; Rooscales et al., 2011) documented digestive tract as a base route of exposure to PAHs in seabirds. Available literature on PAH levels in birds is mainly based on their liver, muscle, fat tissue, eggs, and blood investigation (Kayal and Connell 1995; Nӓf et al. 1992; Paruk et al. 2016; Taniguchi et al. 2009; Troisi et al. 2006). There are limited data on PAH concentrations in seabird lungs. Falkowska et al. (2017) examining PAH levels in the lungs and intestines of herring gull (*Larus argentatus*) reported that, despite the presence of PAHs in aerosol and the fact that they can be inhaled, the introduction with food is more significant for birds. However, the high levels of PAHs in the birds’ lungs from this study point to it being the organ as a similarly important route for birds to be exposed to these compounds. The significant contribution of Naph and its alkylated homologs in the PAH profile in the lungs can be associated with some incomplete combustion processes, exhaust of ships, and evaporation or sublimation from crude oil and petroleum products in the investigated area (Jia and Batterman 2010).

Contact with sufficient concentrations of PAHs may cause a broad spectrum of health effects in seabirds, from altering molecular and physiological processes to modifying hepatic and immune function, increasing physical deformities, reducing reproductive success and growth, and causing acute toxicity in birds covered with oil as an effect of oil spillage or even can lead to death (Albers 2006; Briggs et al. 1997; Bursian et al. 2017; Paruk et al. 2016). Although the acutely lethal effects of oil exposure in birds following catastrophic oil spills are well known (Balseiro et al. 2005; Newman et al. 2000), the PAH chronic exposure among marine birds occupying industrialized coastlines is undocumented. It has been suggested that the potential for mutagenic and carcinogenic effects is greatest among higher molecular weight PAHs (i.e., the 4- to 6-ring compounds and their alkylated forms) (Baird et al. 2007; Lee et al. 2017), but the level of exposure of PAHs at which the adverse effects do not occur in the bird (NOAEL) has not been estimated yet (E.U. 2008). Therefore, it can be stated that the low concentrations of PAHs in the birds from this study, and the particularly low concentrations of the harmful compounds, indicate a negligible exposer of birds to PAHs in the investigated region.

## Conclusion

The study indicates that PAHs are present in the scaup and grebe tissues, and the levels and distribution of these compounds in birds depend mainly on the species specificity. The different metabolic capabilities are the most probable hypothesis to explain the differences in PAH concentrations and profiles between the species. Due to the relatively low concentrations of measured pollutants and the small number of individuals to be investigated, further studies on PAH fate in bird are necessary.

## Electronic supplementary material

ESM 1 (DOCX 21 kb)
