# Supplementary material for: Estimation of native and alkylated polycyclic aromatic hydrocarbons (PAHs) in seabirds from the south coast of the Baltic Sea
Source: Environ Sci Pollut Res Int. 2020 Sep 17;28(4):4366–76. doi: 10.1007/s11356-020-10653-y (PMC7835302; doi:10.1007/s11356-020-10653-y)
Supplement: Supplementary file 1 — (DOCX 21 kb) [file 11356_2020_10653_MOESM1_ESM.docx]

Table 1

| Compound | Retention time (min.) | Reference compound | Quantitation ion (m/z) | Confirmation ions (m/z) |
| --- | --- | --- | --- | --- |
| *Naphthalene-d_8_ (SS-1)*  Naph  C1-Naph  C2-Naph  Acy  *Acenaphthene-d_10_ (SS-2)*  Ace  C3-Naph  Flu  *Phenanthrene-d_10_ (SS-3)*  Phe  *Anthracene-d_10_ (SS-4)*  Ant  C1-DBT  C1-Phe/Ant  C2- DBT  Flt  Pyr  C2- Phe/Ant  B(a)A  *Chrysene-d_12_ (SS-5)*  Chr  B(b)F  B(k)F  B(a)P  *Perylene-d_12_ (SS-6)*  I(cd)P  DB(ah)A  B(ghi)P | 6.37  6.40  7.68  8.66  9.25  9.61  9.68  10.69  11.0  13.96  14.04  14.13  14.19  15.23  16.15  16.91  18.72  19.62  19.95  25.02  25.09  25.19  29.66  29.76  30.90  31.15  34.98  35.13  35.80 | *SS-1*  *SS-1*  *SS-2*  *SS-2*  *SS-2*  *SS-2*  *SS-2*  *SS-3*  *SS-4*  *SS-4*  *SS-4*  *SS-4*  *SS-4*  *SS-4*  *SS-4*  *SS-5*  *SS-5*  *SS-6*  *SS-6*  *SS-6*  *SS-6*  *SS-6*  *SS-6* | 136  128  142  156  152  164  153  170  166  188  178  188  178  198  192  212  202  202  206  228  240  228  252  252  252  264  276  278  276 | 108, 137  127, 129  115, 141  141, 155  150, 151  160, 162  152, 154  155, 169  165, 167  187, 189  176, 179  187, 189  176, 179  197, 199  189, 191  211, 213  200, 203  200, 203  191, 205  226, 229  236, 241  226, 229  250, 253  250, 253  250, 253  132, 260  274, 277  276, 279  274, 277 |

Target GC-MS parameters for PAHs concentrations measurement.

Table 2

Results for blanks analyzed with every set of 9 samples (ng ml^-1^, mean ± SD).

| Compound | Concentration |
| --- | --- |
| Naph  Acy  Ace  Flu  Phe  Ant  Flt  Pyr  B(a)A  Chr  B(b)F  B(k)F  B(a)P  I(cd)P  DB(ah)A  B(ghi)P | 121±31  2.2±1.5  3.5±1.8  17±4.2  29±10  1.8±1.3  9.9±2.7  4.1±1.8  3.5±2.0  5.0±2.9  2.6±1.5  3.2±1.5  2.0±0.9  1.2±0.5  1.5±0.9  1.9±0.5 |

Table 2

Recoveries of the surrogate standards added to the samples and blanks (%, mean ± SD).

| Compound | Recovery |
| --- | --- |
| Naphthalene-d_8_  Acenaphthene-d_10_ Phenanthrene-d_10_ Anthracene-d_10_  Chrysene-d_12_  Perylene-d_12_ | 67±17  98±22  110±29  133±41  158±78  166±92 |
